# ‘What's on the label?’ – the uptake and distribution of alcohol warning labels in Dublin retail stores following delay in policy implementation

**DOI:** 10.1016/j.pmedr.2026.103612

**Published:** 2026-08-29

**Authors:** Lisa Erin Cordos, Saloni Mitra, Evangelia Savvidou, Paula O'Brien, Simone Pettigrew, Eunan McKinney, Daša Kokole

**Affiliations:** a World Health Organization Regional Office for Europe Evidence into Action Alcohol Project (EVID-ACTION) Youth Network, Copenhagen, Denmark; b World Health Organization Technical Advisory Group on Alcohol Labelling, Copenhagen, Denmark; c Melbourne Law School, University of Melbourne, Australia; d The George Institute for Global Health, University of New South Wales, Australia; e World Health Organization Regional Office for Europe, Copenhagen, Denmark; f Department of Health Promotion, CAPHRI Care and Public Health Research Institute, Maastricht University, the Netherlands.

**Keywords:** Alcohol health warning labels, Cancer, Ireland, Implementation, Public health (Alcohol) act

## Abstract

**Objectives:**

In May 2023, under the Public Health (Alcohol) Act 2018, Ireland introduced mandatory labels on alcohol products from May 2026, containing cancer and liver disease warnings, nutritional information and pregnancy pictogram. In July 2025, compliance date was postponed until September 2028, following industry opposition and political pressures. However, anecdotal reports showed occasional early adoption. This study assessed the proportion of alcohol products displaying warning labels shortly after the postponement announcement and five months later.

**Methods:**

Five Dublin stores were selected based on size and geographical representation and visited at two time points: 26 August–8 September 2025 and 30 January–10 February 2026. Products with warning labels were counted across beers, wines, and spirits. Additional product characteristics were recorded.

**Results:**

At the first audit, 6.7% (45/671) of products displayed alcohol warning labels, increasing to 8.3% (54/650) at follow-up, most commonly on wine. Most warning labels were positioned horizontally on the back (93.3%). QR codes featured on 84.4% of products and 13.3% included additional voluntary health-related information.

**Conclusions:**

A limited number of audited alcohol products contained warning labels ahead of the original compliance deadline. Ongoing monitoring is essential to track whether this momentum continues ahead of the September 2028 deadline.

## 1 Introduction

Alcohol consumption is a major contributor to morbidity and mortality across Europe, causing over 200 diseases, cancers and mental health problems, and contributes to injury and violence (Rehm et al., 2017). In the World Health Organization (WHO) European region, 1 in 11 deaths is attributable to alcohol use (World Health Organization, 2024). The International Agency for Research on Cancer has classified alcohol as a ‘Group 1’ carcinogen (*IARC*, 1988), and according to the WHO, no amount of alcohol consumption is considered safe for health and cancer risk (Anderson et al., 2023). However, a 2024 study showed that most Europeans, including Irish, are not aware which specific cancers are alcohol-related (Neufeld et al., 2024). WHO thus underlines the need for clear consumer information, including alcohol warning labels, to reduce harm by increasing public awareness of these health impacts (World Health Organization Regional Office for Europe, 2025).

**Fig. 1:**
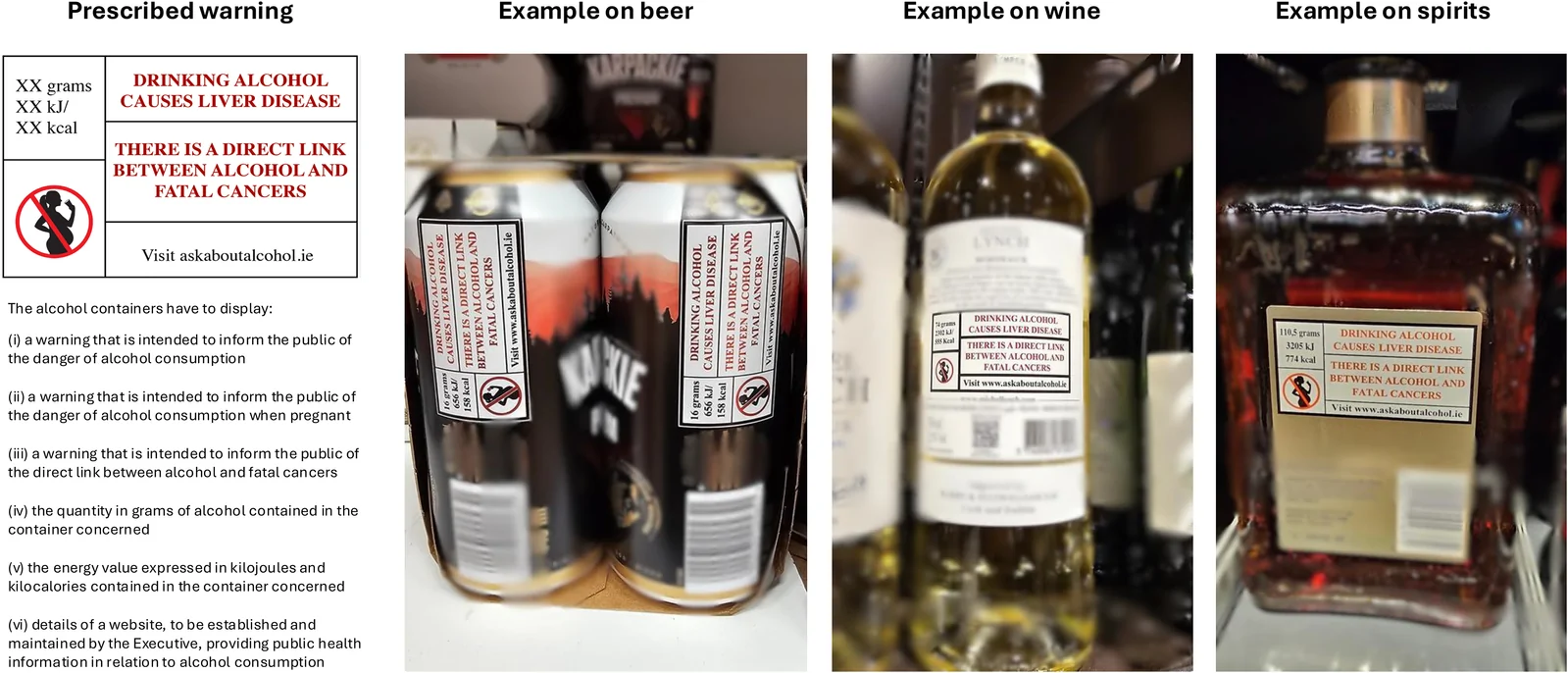
Prescribed alcohol warning label in Ireland and examples of warnings on beer, wine and spirit products as identified in the audit of Dublin stores (August–September 2025 and January–February 2026).

Ireland consistently reports some of the highest levels of alcohol-related harm, ranking in the top 10 European countries with the highest alcohol consumption per capita (World Health Organization Regional Office for Europe, 2024). The Public Health (Alcohol) Act 2018 was introduced to reduce alcohol consumption and the associated harms at a population level, particularly focussing on the prevention of harm to young adults and children (Public Health Alcohol Act, 2018). Labelling of alcohol products with health information is a key measure within the Act, and Ireland was set to become the first country in the world to introduce mandatory multi-component alcohol warning label policy. The labelling regulations were signed in May 2023, with a commencement date of 22nd May 2026 (Murray, 2025). The standard labels were set to include: weight (grams), energy content (kilojoules/kcal), a pregnancy pictogram, two messages stating that alcohol causes liver disease and is linked to fatal cancers, and a signpost to the national alcohol information website (Fig. 1). However, in July 2025, the commencement date was delayed until September 2028, due to intense political and industry opposition, concerning negative impacts on international trade (Alcohol Action Ireland, 2025).

Despite the decision to delay the commencement date, according to informal reports, some alcohol producers and retailers had already begun distributing and displaying products with alcohol warning labels by July 2025, ahead of the original 22 May 2026 commencement date. This activity created the opportunity to examine how the producers and retailers were adopting the warning label and the uptake of the label across various alcohol products, store sizes and geographic locations as part of the implementation of a new labelling measure.

## 2 Methods

### 2.1 Study design and sampling

This audit of alcohol products was conducted in five Dublin stores (three large and two medium-sized) in two periods: the ‘first audit’ (26 August–8 September 2025) and the ‘follow-up audit’ (30 January–10 February 2026). To capture product labelling practices across various retail formats and socioeconomic contexts in County Dublin, the study employed a stratified sampling approach, based on store type and geographic location. Selected stores were of different sizes: large stores with wide product ranges and high footfall (e.g., Tesco Extra and Dunnes Stores) and medium stores (e.g., SuperValu), and distinct geographical areas. These locations included North Dublin City, a region characterised by higher levels of socioeconomic deprivation; South Dublin City, a typically more affluent area; and West Dublin, a rapidly growing suburban area with a mix of socioeconomic profiles. Six stores were initially sampled; permission to conduct auditing was granted from five of the six stores visited at the first audit. The same stores were audited at the follow-up visit.

Beer and wine products with an alcohol by volume (ABV) of over 1.2% and spirits over 15% (as per EU definitions) were sampled. In Medium Stores, all the beer, wine and spirits products meeting the EU definitions were sampled. In Large Stores, we used a random number generator to select two shelf sections for each drink category, separately for each audit. A section was defined as the continuous shelf space bounded by two vertical support columns. Products that were excluded from the study included products with an ABV <1.2%, ciders, pre-mixed drinks, multipacks and products that were locked away or secured behind counters. This study assessed product labelling only and did not involve human participants or animals; therefore did not require ethical approval.

### 2.2 Measures and data collection

Data was collected by the lead study author. At both time points, the total number of unique products in the sampled area was counted, after which each unique product bearing an alcohol warning label was recorded. Additionally, at the first time point, images were captured of each product displaying a warning label and the following information was gathered: type of alcohol product, brand name, country of origin, volume of drink, distributor name, vintage year (if wine product), type of material used for container, price of product, warning location, positioning and sticker format, presence of QR code and presence of additional health information on the label, e.g., “18+” messages.

### 2.3 Statistical analysis

Proportions of products containing alcohol warning labels were calculated for each beverage category, and a two-proportion *Z*-test was conducted to compare the share of labelled products between the two time points. Descriptive statistics (M, N(%)) were calculated for the product characteristics collected at the first audit. Analyses were conducted in Excel.

## 3 Results

Table 1 presents the share of products containing the alcohol warning labels at the two audits. Overall, the share of labelled products increased from 6.7% in the first audit to 8.3% in the follow-up audit, but this difference was not statistically significant (Z = −1.11, *p* = 0.27). Among individual categories, wine had the highest labelling rate in both periods, at 15.6% in the first audit and 13.4% at follow-up, but the small decline was not statistically significant (Z = 0.76, *p* = 0.45). Beer had very low labelling rates in both periods; 0.6% and 2.9% respectively, and the difference did not reach statistical significance (Z = −1.64, *p* = 0.10), likely reflecting the small absolute counts involved. The only statistically significant increase was found for spirits, rising from 0.9% in the first audit to 4.7% at follow-up (Z = −2.44, *p* = 0.02).

**Table 1:** Numbers and proportions of sampled products (total, beer, wine and spirits) containing new alcohol health warning labels in Dublin, Ireland across the two measurement points (August–September 2025 and January–February 2026).

	First Audit			Follow-up Audit			
	N labelled products	N total	%	N labelled products	N total	%	Sig. a
Total	45	671	6.7	54	650	8.3	0.27
Beer	1	178	0.6	4	139	2.9	0.10
Wine	42	269	15.6	40	299	13.4	0.45
Spirit	2	224	0.9	10	212	4.7	0.02

a A two-proportion *Z*-test was conducted to compare the share of labelled products between the first audit (26 August – 8 September) and the follow-up audit (30 January – 10 February).

At the first audit, further details on the characteristics of the labels and the labelled products were assessed. Labelled product containers were mostly glass (97.8%) and were mostly 750 ml in volume (91.1%). The remaining sizes were two 500 ml, one 700 ml, and three 200 ml containers in a joint package. Most of the labels were positioned horizontally on the back of the product (93.3%, 42 products), with the remainder positioned vertically on the side (6.7%, 3 products). In 95.6% of cases (43 products), the health warning was integrated into the product label, only 4.4% (2 products) utilised separate stickers. France was the most represented country among labelled wine products, accounting for 28.9% of the total (13 products), followed by Australia at 22.2% (10 products), Spain at 13.3% (6 products). Chile and Italy each accounted for 11.1% (5 products each), while New Zealand represented 6.7% (3 products). Argentina, Poland, and Portugal each contributed a single labelled product, each making up 2.2% of the total. Overall, there were no deviations from the legislated design. Additionally, thirty-eight products (84.4%) also contained a QR code, and six (13.3%) products contained additional health warnings voluntarily included by the producers, such as “drink responsibly” messages, “18+” graphics and extra pregnancy pictograms.

Among the identified 42 labelled wine products, the mean price, where recorded, was €18.28. The 2024 vintage was the most common, accounting for 45.2% of labelled wines (19 products), followed by 2023 at 26.2% (11 products), 2025 vintage at 4.8% (2 products), and the 2021 and 2022 vintages each contributing a single product at 2.4% apiece. Vintage information was not visible from the recorded images for 19.0% of labelled wines (8 products).

## 4 Discussion

This study found that about one in 15 alcohol products across five Dublin stores already complied with mandatory labelling requirements at least nine months ahead of the original May 2026 deadline, rising from 6.7% at the first audit in August 2025 to 8.3% in January 2026.

The majority of labelled products were wines, possibly reflecting that producers already had to comply with Regulation (EU) 2021/2117(Regulation (EU) 2021/2117 of the European Parliament and of the Council, 2021), requiring wines to list ingredients and nutritional information, making it easier to make other label changes as well. The large proportion of wine products displaying warning labels may also be due to routine annual vintage label updates, as well as the centralised structure of wine importation in Ireland, where importers are well-placed to apply labels and comply with legislation, even though liability under the Act falls on the seller.

Anecdotal industry reports suggests that some producers chose to act early for practical or commercial reasons, such as long shipping times and avoiding supply disruption downstream (*The New Zealand Herald*, 2025). Celebrity wine brands also adopted labels voluntarily before the compliance date and there was no report of negative impact on sales (*The Sunday Times,* 2025), although labels were removed after compliance date was postponed to 2028 (O'Donoghue, 2026). It is not clear, however, whether early uptake signals support for the label or whether it reflects practical considerations, rather than endorsement of the measure. Many alcohol producers opposed the regulation, citing costs and trading impacts through the EU TRIS (Technical Regulation Information System) notification process, an EU mechanism designed to prevent unfair trade barriers (Cott et al., 2025).

The increase in spirits labelling from 0.9% to 4.7% may reflect individual decisions by specific distributors rather than any broader shift in the sector and the consistently low labelling rates for beer across both periods are consistent with the strong opposition to the regulation from major brewing companies (*Follow the Money,* 2026) and rapid product turnover. Therefore, any labelling changes were likely to be completed nearer to the compliance date.

The Food and Drink Federation in the UK has noted that a 24-month transition period is sufficient for industry to adapt to new labelling requirements (The Food and Drink Federation, 2026), matching the EU's two-year transition period for Regulation 2021/2117. Ireland's 36-month period should thus have been more than sufficient for legislation compliance. The relatively limited uptake in our study suggests that many producers were delaying labelling to as close to the commencement date as possible, reflecting the broader pattern of industry resistance to implement mandatory health warning labels.

Finally, a recent cross-sectional study showed that early exposure to warning labels in the Irish population have resulted in 32.5% respondents noticing them (Kokole et al., 2026), suggesting that even if the proportion of labelled products was not high, the labels were still disproportionally noticed. Two thirds of participants noticing the labels reported labels informed them about alcohol-related health risks and a quarter decided to buy less or no alcohol on that shopping trip (Kokole et al., 2026).

This study has a number of limitations. Firstly, while audits were carried out in major retail outlets in one city that are reflective of the wider off-trade Irish market, there may be a variance in other regional or rural retail settings. Secondly, the two audit periods five months apart fall in different seasons, potentially affecting the range of products available on shelves, making direct comparisons less straightforward. Thirdly, prices were not recorded for all products captured. As such, an average was obtained from the available data. Finally, the study covered beer, wine and spirits only, excluding other product categories such as cider and premixed drinks, which are also required to carry warning labels. For feasibility reasons, the product characteristics were only collected at the first audit, and future research could continue monitoring these alongside label prevalence.

## 5 Conclusion

A small but growing proportion of alcohol products on Dublin shelves carried the alcohol warning labels required under the Public Health (Alcohol) Act 2018, rising from 6.7% in late summer 2025 to 8.3% five months later. Uptake was concentrated mainly among wines. With compliance postponed to September 2028, it remains to be seen whether this momentum will continue or slow. Ongoing monitoring will be essential to track how uptake develops across different product categories and retail contexts as the revised deadline draws closer.

## Availability of data and materials

The dataset generated and analysed during the current study is available in the Figshare repository: https://doi.org/10.6084/m9.figshare.33227076.

## CRediT authorship contribution statement

**Lisa Erin Cordos:** Writing – original draft, Methodology, Investigation, Formal analysis. **Saloni Mitra:** Writing – review & editing, Methodology, Formal analysis. **Evangelia Savvidou:** Writing – review & editing, Methodology, Formal analysis. **Paula O'Brien:** Writing – review & editing, Methodology, Conceptualization. **Simone Pettigrew:** Writing – review & editing, Methodology, Conceptualization. **Eunan McKinney:** Writing – review & editing, Methodology, Conceptualization. **Daša Kokole:** Writing – review & editing, Supervision, Methodology, Conceptualization.

## Ethics declaration

The author(s) declare(s) that the study does not involve humans nor animal subjects.

## Funding

This research did not receive any specific grant from funding agencies in the public, commercial, or not-for-profit sectors.

## Declaration of competing interest

None declared. Lisa Erin Cordos, Saloni Mitra and Evangelia Savvidou are members of World Health Organization (WHO)’s EVID-ACTION Youth Network. Paula O'Brien, Simone Pettigrew and Eunan McKinney are members of WHO Technical Advisory Group on Alcohol Labelling. Daša Kokole is a WHO consultant. The authors alone are responsible for the views expressed here and these do not necessarily represent the decisions or the stated policy of WHO.
